# Glans ischemia in a 7-month-old boy who underwent correction of megaprepuce: case report and review of the literature

**DOI:** 10.3389/fped.2024.1460717

**Published:** 2025-02-27

**Authors:** Susanne Kraske, Fanny Müller, Vera Schellerer

**Affiliations:** Department of Pediatric Surgery, University Medicine Greifswald, Greifswald, Germany

**Keywords:** glans ischemia, megaprepuce, dorsal penile nerve block, electrocautery, dressings, pentoxifylline, heparin

## Abstract

Acute glans ischemia is a severe complication that can occur after penile surgery, such as circumcision, hypospadias repair, or disassembly of the corpora in epispadias. This rare condition is described in the current literature, especially after circumcision, although it is the less invasive procedure among those previously mentioned. We present a case of 7-month-old boy who developed glans ischemia after correction of megaprepuce. The condition was treated by intravenous unfractionated heparin. In our case, the complication of ischemia was complete dehiscence of the suture line at the coronal sulcus. Glans penis recovered completely and secondary intention wound healing occurred within 4 weeks. We reviewed the literature over the past 43 years (1981–2023) to describe the current options of surveillance, treatment, and outcome of glans ischemia after penile surgery in boys.

## Introduction

Several techniques have been described for the correction. At our institution, the surgical technique is performed according to Hirsch et al. or Shalaby and Cascio, with few modifications, with complete degloving of the penile shaft combined with a VY-plasty at the penoscrotal junction (1, 2). The inner prepuce is resected subtotally and not used for penile shaft coverage. Although it is an invasive operation, glans ischemia was not previously described in this procedure. The current literature discusses that ischemic complications in penile surgery may result from local anesthetics with or without vasoconstrictor agents such as epinephrine, vasospasm induced by dorsal penile nerve block (DNPB) and their techniques (e.g., ringblock vs. bilateral injections), microtrauma caused by cautery, circular bandages, increased local pressure due to edema and local hematoma, or the use of tourniquets to achieve blood-free surgical fields (3–15). All these conditions temporarily reduce blood flow and can lead to transient ischemia or, if long-lasting, to gangrene.

**Figure 1:**
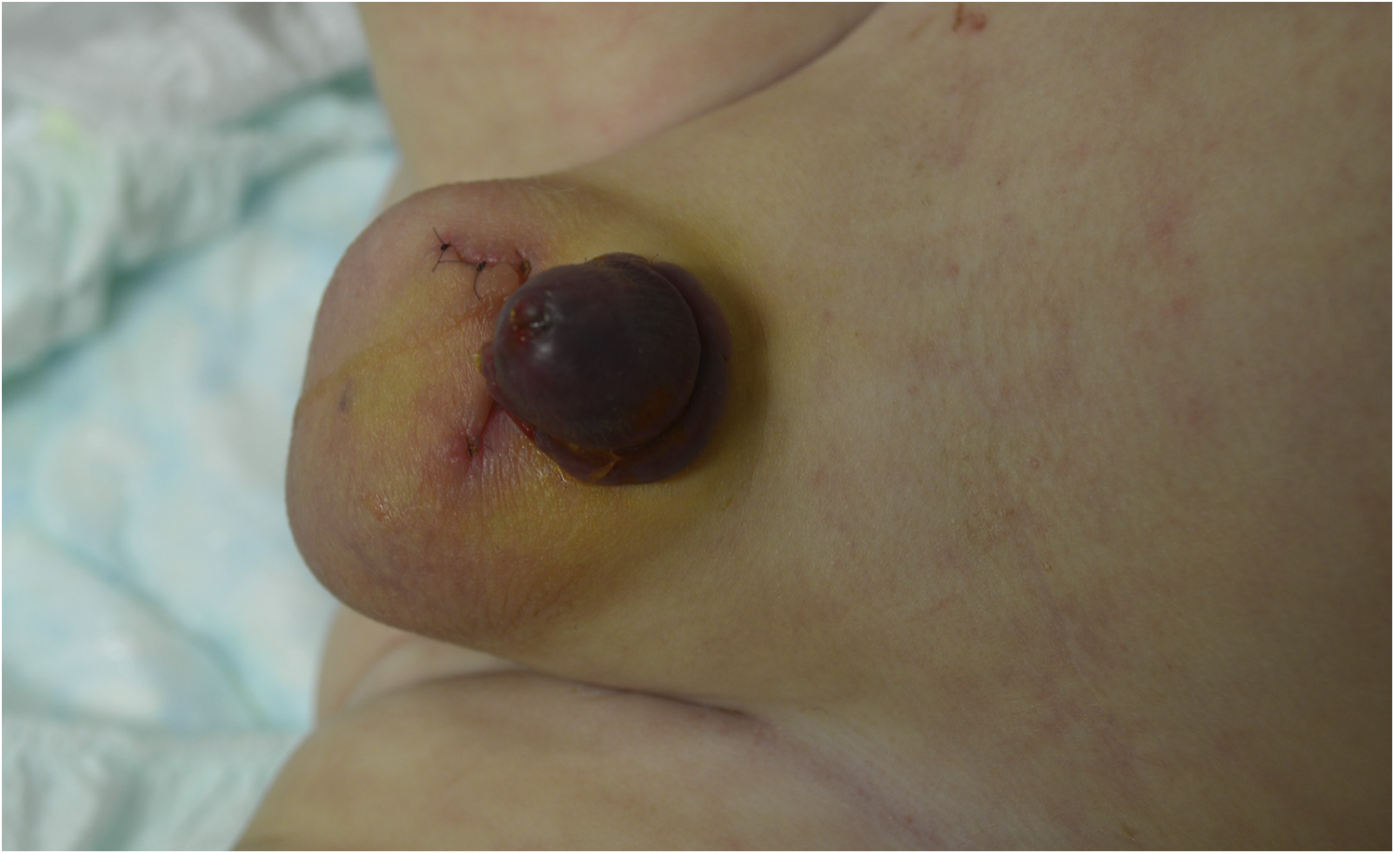
Appearance of the glans after removing the bandage on postoperative day 4.

**Figure 2:**
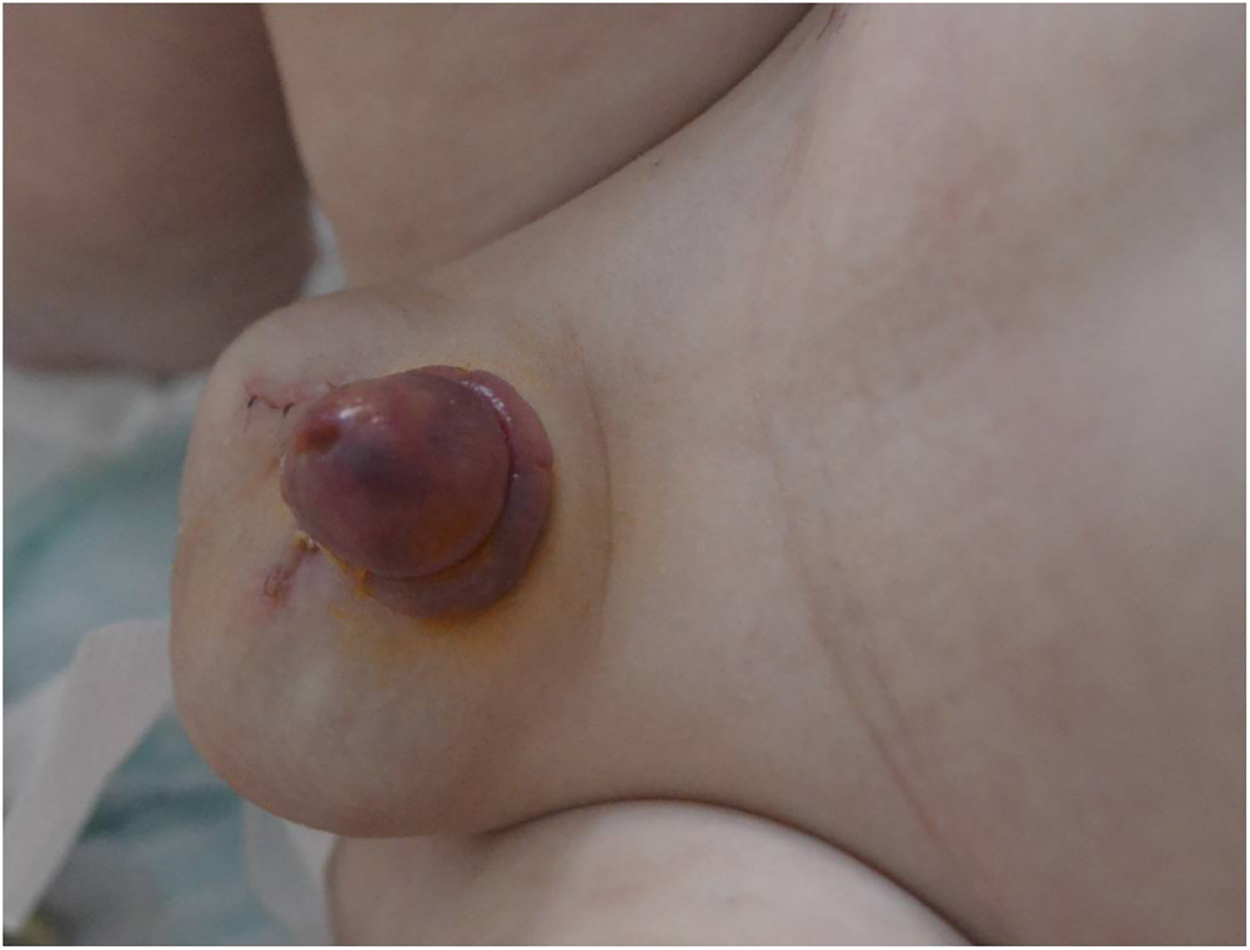
Appearance of the glans on day 4 after initiating IV heparin treatment.

**Figure 3:**
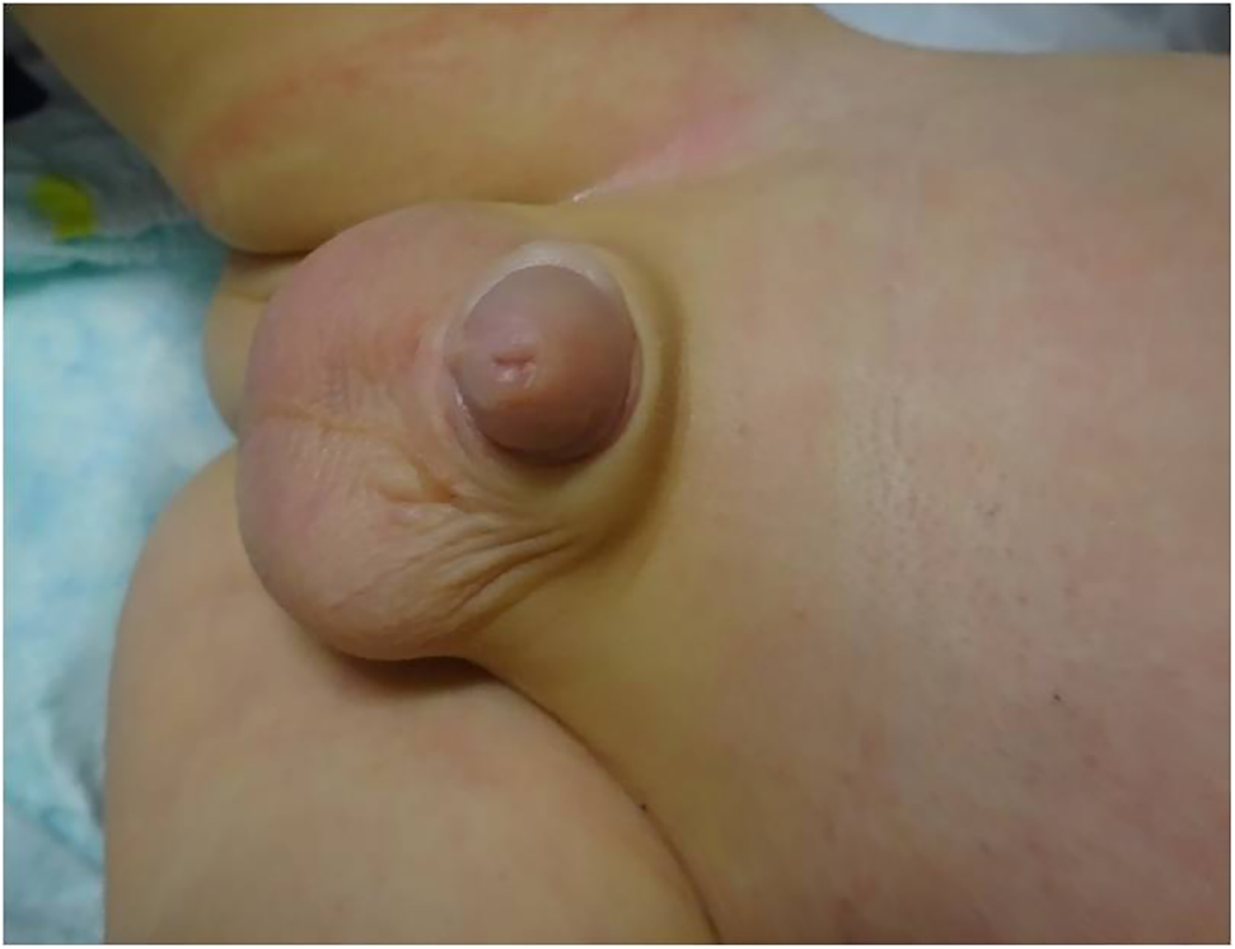
Penile appearance 4 weeks postoperatively.

In the present case, renewing of dressing at day 3 postoperatively potentially led to glans ischemia. Unfractionated heparin (UFH) was used for treatment according to the current reviewed literature.

## Case report

A 7-month-old boy (at 5 months of adjusted age) was referred to our department for the correction of megaprepuce. He was born preterm at 28 weeks and 4 days of gestation with a birth weight of 980 g. Urinary tract infections (UTIs) occurred due to urinary retention under the foreskin and his parents expressed the foreskin from time to time to empty residual urine completely. The child exhibited postponement of micturition, which raised the parents’ concerns due to a history of infections and the inconspicuous appearance of the penis. Surgery was performed to prevent further UTIs and to treat voiding postponements. A preoperative ultrasound examination revealed a normal upper and lower urinary tract and intraoperative cystoscopy showed no signs of urethral valves. The ureteric orifices were cone-shaped and located at the trigone.

The correction of megaprepuce was performed under general anesthesia and caudal epidural block with 2% ropivacaine. First, a small ventral slit of the foreskin was made, extended only to the point where the foreskin could be fully retracted. After inspecting the glans, a circumferential incision was made parallel to the coronal sulcus, leaving a 4 mm mucosal collar intact. The penile skin was incised ventrally along the penile raphe, degloved to the penopubic level dorsally and to the level of penoscrotal junction ventrally, leading to penile straightening. The inner prepuce was partially resected. The skin was mobilized by VY-plasty at the ventral base of the penis to gain length of the ventral skin to cover the penile shaft at this site and to remodel the penoscrotal junction. No temporary tourniquet was applied. At the end of the surgical procedure, a transurethral catheter and loose padding bandage were placed. On the third postoperative day, the bandage was removed, revealing well-healing tissue with smooth, rosy skin. The boy’s discharge was scheduled for the following day. However, as the boy passed soft to fluid stools several times in the afternoon, the pediatric surgeon on the night shift applied a circular bandage. The patient developed a subfebrile temperature of 38.8°C approximately 3 h after the bandage was renewed. The boy experienced interrupted and fitful sleep. The next day, the circular dressing was removed, revealing a black-colored glans and inner preputial rim with a dry surface.

Within 2 h of the bandage being removed, no visual improvement of the glans was observed.

The patient’s body temperature returned to normal 4 h after the dressing was removed. A urine probe showed no signs of UTI. We removed the transurethral catheter to avoid any pressure on the corpus spongiosum whose distal end is the glans penis. Further treatment opportunities were discussed with pediatricians.

Even if no uniform treatment guidelines exist, the literature reports different options for medical treatment in children (Table 1), such as therapy with pentoxifylline (PTX) or heparin (5, 6, 8, 17–22).

**Table 1:** Summary of the retrieved articles for the treatment of post-operative glans ischemia.

Reference	Number of patients/operation	Age at operation	Suspected cause/surgical technique	Therapy	Onset of complains	Onset of therapy post operative	Duration of therapy	Complications
Akram et al. (3)	1 circumcision	5 yrs.	Incomplete circumcision leading to paraphimosis	Antibiotics (ceftriaxone and clindamycin) amputation of the gangrenous portion of the penis suprapubic cystostomy delayed penile reconstruction	7 days	15 days	10 days	Partial loss of glans penis
Aminsharifi et al. (16)	2 circumcision	Case 1 3 yrs. Case 2 4 yrs.	No expectations/sleeve circumcision in both cases	10% testosterone cream twice a day in both cases	Not mentioned	7 days 3 days	4 wk. 4 wk.	Hypospadia None
Aslan et al. (17)	1 circumcision	11 yrs.	Circumferential DPNB 0.1% xylocaine firm bandage	i.v. PTX 10 mg/kg per day divided into four equal doses	24 h.	72 h.	5 days	None
Assaf et al. (4)	1 circumcision	6 days	Monopolar electrocautery	Surgical debridement meatoplasty and phalloplasty at 4 months and 11 months	6 days	8 days	Immediate surgical procedure	Loss of glans recurrent meatal stenosis
Burke et al. (5)	1 circumcision	18 yrs.	DPNB, midline, 8 ml of 0.75% ropivacaine, further 2 ml injected s.c. around frenulum, bipolar electrocautery	Dalteparin 15.000 IU iloprost infusion (0.5 to 2 µg/h)	“Shortly after” […] operation palor of glans penis, followed by loss of sensation	8 h.	43 h.	None
Carlaw et al. (6)	1 circumcision	11 yrs.	DPNB (10 ml of 0.5% bupivacaine without epinephrine)	2% nitroglycerin ointment twice a day oral pentoxifylline 400 mg three times a day epidural anesthesia 0.2% ropivacaine	4 h.	7 h.	11 days 14 days 3 days	None
Codrich et al. (18)	2 circumcision suspected causes after in 2 cases were discussed	Case 1 8 yrs Case 2 10 yrs	General anaesthesia, DPNB with mepivacaine monopolar electrocautery no compressive bandage general sedation, spinal anesthesia bipolar electrocautery no compressive bandage	Case 1 Subcutaneous enoxaparin 2,000 IU once a day nitric oxide ointment once a day Case 2 Subcutaneous enoxaparin 3,000 IU once a day	6 h. 4 h.	Immediately immediately	5 days 1 week 5 days	None None
Devarakonda et al. (7)	1 redo Hypospadia	4 yrs.	Penile tourniquet, total ischemic time was 90 min.	Epidural analgesia with 7 ml of 0.25% levobupivacaine, and a continuous infusion of 0.125% levobupivacaine epidural	Immediate after Tourniquet release	Immediately	48 h	None
Efe et al. (8)	1 circumcision	7 yrs.	DPNB 0.1% xylocaine+ephedrine	i.v. PTX 10 mg/kg divided into four equal doses	24 h.	24 h.	6 days	None
Garrido-Abad et al. (10)	1 circumcision	3 yrs.	2 sub-pubic symmetrical 2 ml injections of 1% mepivacaine without adrenalin, 1 cm lateral to the midline	general anesthesia, caudalblock (bupivacaine 0.25% 1 ml/kg) surgical suture liberation topical nitroglycerin, gentamicin oral pentoxifylline 60 mg/8 h (3.3 mg/kg)	48 h.	48 h.	Immediately Immediately 7 days 7 days	None
Gnatzy et al. (11)	1 circumcision	16 yrs.	DPNB (15 ml of 0.25% bupivacaine each side)	Immediate angiographv intraarterial spasmolysis (5 µg alprostadil and 150 µg nitroglycerine sequentially) systemic therapv sildenafil (1 mg/kg orally once a day), L-arginine (0.1 mg/kg/h) unfractionated heparin (15 IU/kg/hour, up to 20 IU/kg/hour	24 h.	24 h.	Immediate 3 days	None
Hasegan et al. (25)	1 circumcision	17 yrs.	No expectations Surgery under/with: General anesthesia DPNB 10 ml of 0.5% bupivacaine Bipolar electrocautery No circular dressing	Enoxaparin 4,000 IU daily local patch of 25 mg of glyceryl trinitrate, topical nitroglycerin 2% ointment twice daily amoxicillin continuous epidural infusion of 0,2% ropivacaine i.v. PTX 10 mg/kg 3 times a day alprostadil 20 mg twice daily HBO following unsuccessful previous therapy	5 h.	Immediately Started at day 2 Started at day 2 Started at day 2 Started at day 3 Started at day 5	5 days 5 days 5 days 5 days 5 days 1 day 5 daily sessions	None
Kaplanian et al. (28)	1 circumcision	9 yrs.	No expectations DPNB with 3 ml of 0.5% plain bupivacaine injected on each side lateral to the midline	caudal epidural block	4 h.	Immediately	Once	None
Karaguzel et al. (19)	1 circumcision	4 yrs.	No expectations bandage for 24 h. no DPNB bipolar electrocautery	i.v. PTX 10 mg/kg divided into four equal doses	48 h.	48 h	6 days	None
Migliorini et al. (26)	1 circumcision	24 yrs.	DPNB 10 ml of 2% mepivacain monopolar electrocauterization moderately compressive wound dressing	intracavernosal 2.5 µg PGE1 injection PTX with gradually augmented dossage PTX 100 mg i.v two times a day PTX 200 mg i.v two times a day PTX 250 mg i.v two times a PTX 400 mg orally HBOT Amoxicillin orally	24 h.	24 h.	Once 15 days: 48 h. 48 h. 72 h. 8 days 7 days 8 days	None
Mirnia et al. (20)	1 circumcision	7 days	Circumcision with ring-methode DPNB 0,1% lidocaine	Antibiotics, then discharged and admitted at specialized hospital: oxacillin and amikacin, mupirocin ointment p.o. PTX (10 mg/kg/day)	48 h.	48 h. 96 h.	2 days 12 days 10 days	Urethral fistula
Mittino et al. (30)	1 circumcision	33 yrs.	lidocaine 2% and carbocaine	Reoperation warm saline, renewing of the suture, a dorsal cutaneous incision was performed Medication: Enoxaparin 2,000 IE s.c. daily ASS 100 mg daily	24 h.	48 h.	Immediately 20 days 20 days	None
Ozzeybek et al. (27)	1 circumcision	11 yrs.	No expectations	Reoperation sutures were removed Dorsal penile nerve block (3 ml or 0.25% bupivacaine) Medication: intracavernous glycerol trinitrate (3 mg/kg, 2 ml) continuous epidural sympathetic block (Bupivacaine 0.0625%) i.v. Rheomacrodex 0.3 ml/kg/h	72 h.	72 h.	Immediately 5 days 6 days	None
Palinrungi et al. (12)	1 circumcision	8 yrs.	DPNB with accidental addition of epinephrine to Lidocaine	Wound crust cleansing saline compress locally	1 h.	24 h.	7 days	None
Pepe et al. (13)	1 circumcision	20 yrs.	Volume of DPNB 1% mepivacaine Technique of DPNB Bandage for 24 h.	HBO Antiplatatelet: aspirin 330 mg daily Levofloxacin 500 mg prednisone 25 mg daily	24 h	5 days	10 days 30 days 15 days 10 days	None
Polak et al. (14)	2 circumcision	Case 1 8 days Case 2 8 days	Amputation of mid-glans and urethra increased bleeding tight homeostatic bandage left for 72 h	Anastomosis of the amputated glans and urethra over a catheter HBO, bilateral preventive myringotomy Removal of Bandage observation HBOT, bilateral preventive myringotomy	Immediately 72 h.	Immediately 72 h. 5 days	7 days 48 h. 7 days	None Loss of glans penis
Soltani et al. (21)	1 circumcision	4 yrs.	Glans amputation at circumcision	Immediate replantation 200 mg PTX twice a day, orally	Immediate Operation HBO	Immediate	7 days	None
Sterenberg et al. (23)	1 circumcision	7 days	bandage	No specific therapy observation	72 h	Removal of bandage		Loss of glans penis
Uzun et al. (15)	1 circumcision	7 yrs.	Monopolar cauterization caused burn injury	HBO	Immediately	Several hrs.	n. m.	Loss of glans and partial loss of urethra leading to hypospadia
Zvizdic et al. (22)	1 circumcision	6 mon.	DPNB Lidocaine 2% Bandage	Enoxaparin 1.25 mg/kg s.c. DHT ointment 2,5% twice a day	Several hrs.	24 h.	5 days	None
Fahmy et al. (9)	prospective study: 3,382 post- circumcision in 23 of the patients penile ischemia occured	From 4 weeks to 18 yrs. of age	74% associated with the use of monopolar electrocautery 52.2% associated with compressive wound dressing 73.9% associated with inexperienced hysicians	Combination of: hyperbaric oxygen therapy, pentoxifylline with 10 mg/kg/day divided into three equal doses, third generation cephalosporin antibiotic (Cefotaxime)	2 groups: Therapy within 24 h. and >24hrs.	Divided into 2 groups: within 24 h. and >24hrs. Patients managed at tirst 24 h had better outcomes	10 to 21 days	Glanular/urethral loss: 2 hypospadias 3 Partial penile loss 6 Complete penile loss 2
Tasci et al. (24)	24 patients with penile necrosis after circumcision between 2003 and 2013; retrospective study	20 days to 16 yrs. Median 5 + 3.7 yrs.	Monopolar cautery (10/24, 41.6%) post-circumcision (8/24, 33.3%) dressing (3/24, 12.5%) local anesthetic agent (2/24, 8.3%)	Surgical revision (15/24, 62.5%) HBOT (6/24, 25%) conservative approach (2/24 8.3%) HBOT plus surgical intervention (1 24, 4.1%)	Not mentioned	1 h. to 21 days		Reconstructive surgery: skin graft in 9 patients partial amputation and skin graft in 3 urethroplasty and skin graft in 3

DHT, dihydrotestosterone; DPNB, dorsal penile nerve block; PTX, pentoxifylline; HBO, hyperbaric oxygen therapy; Hrs., hours; mon., months; yrs., Years; n.m., not mentioned.

In contrast to PTX, UFH is a widely used and safe therapy for many conditions in infants. In contrast to subcutaneous low-molecular-weight heparin (LMWH), UFH is well adjustable. We therefore decided to begin a continuous intravenous infusion of UFH with 10 IE/kg/h as low-dose therapy. Anticoagulant therapy was adjusted based on partial thromboplastin time (PTT), with values set above the baseline normal range. The first PTT control was made 6 h after the low-dose heparin infusion was started.

Doppler ultrasound was performed before heparin therapy and 24 h after. In each examination, neither abnormal flow pattern in the dorsal penile artery nor thrombosis of the corpora cavernosa was seen. Approximately 4 h after heparin therapy was started, reddish spots appeared on the dark-colored glans. Within 24 h, the glans became responsive, with the initial livid discoloration that gradually becoming increasingly rosy. No modifications or additions to previous therapy were implemented due to the continuous visible improvement. The glans had an almost pink appearance in all areas on day 4 of the continuous intravenous heparin administration. At this point, the therapy was ended, and the boy remained under surveillance as an inpatient for another 24 h. The patient was discharged without any medication.

In our case, as a complication of ischemic conditions, complete dehiscence of the suture line at the coronal sulcus occurred; however, the wound healed completely secondarily within another 4 weeks. Despite this complication, there were no signs of gangrene, such as blistering or shedding, at the surface of the glans. In the patient’s outpatient visits at 4 weeks and 6 months postoperatively, no further deterioration was noticed. We observed good cosmesis with a completely recovered glans penis and no signs of meatal stenosis, hypospadias, or concealed penis.

## Methods and review of the literature

According to the Preferred Reporting Item for Systematic Reviews and Meta-Analyses (PRISMA), a systematic search of English-language full-text articles was made on the PubMed Database. The keywords were “glans ischemia” (73 results), “glans necrosis” (208 results), and “glans ischemia after circumcision” (26 results). Duplicates and articles that were not concerned with glans ischemia after penile surgery in children or adolescents were removed. We then checked all the reference lists of the included articles. In total, 27 articles were analyzed (25 case reports, 1 retrospective study, and 1 prospective study), due to surgical procedure, onset of perfusion disturbances, patients’ age, therapeutic measures and duration, monitoring of therapy, and outcome (Table 1).

## Results

No guidelines exist for the treatment of glans ischemia. As shown in Table 1, the reviewed literature reports on the duration of therapy and outcome as well as different therapeutic strategies. Such strategies are systemic or local vasodilatation, local spasmolysis, anticoagulation, or rheological changes. Hyperbaric oxygen therapy (HBOT) for better oxygen administration is also described in detail. Treatment with heparin (LMWH and UFH) or PTX for systemic vasodilatation, anticoagulation, and rheological changes was described in nine reviewed articles (5, 6, 8, 17–22).

The authors reported surgical procedures alone in five cases or only surveillance in one case (3, 4, 12, 14, 15, 23).

HBOT alone was described in detail and within the retrospective study of 24 cases from Tasci et al. (24, 25). Antibiotics were administered in seven of the reported cases (3, 9, 10, 13, 20, 25, 26). In three cases, epidural anesthesia was used for sympathicolysis to treat glans ischemia (7, 27, 28).

Neither a method for surveillance of therapy nor for the initial assessment of glans ischemia was described in the reviewed literature. Initial and follow-up grayscale, color Doppler ultrasound, and D-dimer levels were reported (8, 11, 19, 29). However, only Efe et al. described pathologic findings in grayscale ultrasound, including thickening of the epithelial layer with normal penile and glanular blood flow (8, 30). In contrast to those findings, Barnes et al. revealed a lack of vascularity in the glans on color Doppler imaging and hypoechoic glans tissues in high frequency grayscale ultrasound in a 2-week-old male infant who developed glans ischemia after circumcision. In the same study, an ultrasound scan in a patient of the same age showed no such pathologies after an uneventful circumcision (29).

Gnatzy et al. reported a case of glans ischemia in a 16-year-old boy treated by angiography with local spasmolysis. However, neither vasospasm nor blood flow abnormities were found on angiography (11). No evidence was found correlating glans ischemia with detectable changes in blood flow abnormalities on color Doppler ultrasound or angiography.

Efe et al. also describe elevated D-dimer levels of 2.57 mg/L in a 7-year-old boy with severe ischemia of the glans penis (8), whereas Gnatzy et al. and Karaguzel et al. found normal D-Dimer levels in their patients (11, 19).

There are inconsistent findings in the assessment of glans ischemia and its probable causes. Post-circumcision findings suggest that ischemia may result from specific surgical factors, such as local anesthesia in DPNB, the volume used, with or without vasoconstrictive agents, tight bandages, hemorrhage, a tight suture line, or monopolar cauterization, which may cause thermal injury or vasospasm due to the electric current passing through the small penile diameter. However, a review of the current literature does not provide clear evidence correlating glans ischemia with specific steps in the surgical procedure. For example, Codrich et al. compared two cases from their institution where glans ischemia occurred after circumcision in boys aged 8 and 10 years (18). One operation was performed under general anesthesia with a dorsal penile nerve block, using monopolar cauterization, while the other was under general anesthesia with an additional caudal block and bipolar cauterization. Both surgeries involved the same suturing technique, with no dressing applied. In both cases, transient glans ischemia occurred.

However, within the reviewed literature, severe complications including partial penile loss are consistently reported, especially after a delay in the beginning of treatment (3, 4, 9, 14–16, 20, 23, 24).

## Conclusion

At our institution, only bipolar cautery is used during penile operations such as hypospadias surgery, correction of buried penis, and megaprepuce. Surgery is performed under general anesthesia with an additional caudal block with 0.2% ropivacaine or dorsal penile nerve block. Dressings are placed with care only in the operating room using the sandwich technique or as padding bandages. It is mandatory that the glans is visible despite the bandage for postoperative evaluation. After the dressing is removed, a new bandage should be avoided. First, children get scared bandages are changed. Furthermore, there is no proven benefit of changing the dressing. In the present case, placing a circular dressing potentially led to glans ischemia. After treatment with intravenous UFH, the glans recovered completely. The question of whether reperfusion would occur spontaneously without intervention remains uncertain. In our case, early therapy was initiated based on findings in the literature, which highlight that complications are common after treatment is delayed.

Long term follow-up after transient glans ischemia is necessary to monitor for late complications, such as meatal stenosis.

## Data Availability

The raw data supporting the conclusions of this article will be made available by the authors, without undue reservation.
